# Pulmonary Arteriovenous Fistulae After Fontan Operation: Incidence, Clinical Characteristics, and Impact on All-Cause Mortality

**DOI:** 10.3389/fped.2022.713219

**Published:** 2022-06-09

**Authors:** Hideo Ohuchi, Aki Mori, Michikazu Nakai, Kazuto Fujimoto, Toru Iwasa, Heima Sakaguchi, Kenichi Kurosaki, Isao Shiraishi

**Affiliations:** ^1^Department of Pediatric Cardiology, National Cerebral and Cardiovascular Center, Suita, Japan; ^2^Adult Congenital Heart Disease, National Cerebral and Cardiovascular Center, Suita, Japan; ^3^Department of Medical and Health Information Management, National Cerebral and Cardiovascular Center, Suita, Japan

**Keywords:** Fontan operation, pulmonary arteriovenous fistulae, arterial oxygen saturation, heart failure, mortality

## Abstract

**Background:**

The Fontan operation is a surgical procedure used in children with univentricular hearts. Pulmonary arteriovenous fistulae (PAVF) is a major complication after a Fontan operation. However, the incidence and related clinical pathophysiology of PAVF remain unclear.

**Purpose:**

This study aimed to clarify the incidence of PAVF, its clinical characteristics, and its influence on all-cause mortality.

**Methods and Results:**

We serially assessed the presence of PAVF using pulmonary artery angiography and/or contrast echocardiography during catheterization in 391 consecutive patients who underwent the Fontan procedure and compared the results with the Fontan pathophysiology and all-cause mortality. PAVF developed in 36 patients (9.2%), including 30 diffuse- and six discrete-PAVF types. The PAVF-free rates at 1, 5, 10, 15, 20, and ≥25 years after Fontan operation were 97, 96, 93, 88, 87, and 83%, respectively. The mean arterial blood oxygen saturation (SaO_2_) in patients with diffuse PAVF at each corresponding postoperative stage were 90, 91, 91, 91, 89, and 88%, respectively, indicating lower SaO_2_ levels than those in patients without PAVF (all *p* < 0.01). However, there was no difference in the SaO_2_ levels between patients with discrete PAVF and those without PAVF. During a median follow-up period of 2.9 years after the last catheterization, 31 patients, including 12 patients with PAVF, died. Patients with PAVF, especially those with diffuse PAVF, had a higher mortality rate (*p* = 0.01) than those without PAVF (hazard ratio: 3.6, 95% confidence interval: 1.6–7.8, *p* = 0.0026).

**Conclusion:**

Patients who underwent Fontan surgery had an increased incidence of PAVF as they aged. Discrete PAVF did not influence SaO_2_ or mortality, whereas the presence of diffuse PAVF caused hypoxia and was associated with all-cause mortality.

## Introduction

The Fontan procedure is a palliative surgery that aims to improve survival in infants born with a functionally univentricular circulation. Although most patients who undergo the Fontan operation can survive into adulthood, the postoperative morbidity and mortality rates remain high, probably because of the longstanding unique hemodynamics (1). Of the well-known post-Fontan complications, such as heart failure, arrhythmias, and protein-losing enteropathy, pulmonary arteriovenous fistulae (PAVF) is a major and serious complication (2, 3). The close association of maldistribution or lack of hepatic venous flow to the pulmonary arteries bilaterally has been repeatedly demonstrated; therefore, the “hepatic factor” hypothesis has been proposed as a cause of PAVF (2, 3). However, the mechanism of PAVF formation or how it causes damage remains unknown. Furthermore, PAVF-related clinical characteristics, such as its incidence and/or impact on prognosis, have not been well-described because of difficulties in detecting PAVF, especially incipient PAVF, as there is no visible influence on Fontan hemodynamics, including arterial oxygen saturation (4). Thus, only a few studies have addressed the long-term impact of PAVF on Fontan survivors. Therefore, a serial comprehensive assessment of the pathophysiology of the Fontan circulation gave us a unique opportunity to clarify its impact (5). Accordingly, the aims of the present study were: (1) to clarify the incidence of PAVF; and (2) to assess the association between PAVF and the Fontan pathophysiology, including all-cause mortality.

## Materials and Methods

### Participants

Overall, 485 patients underwent the Fontan operation between October 1979 and February 2018 at our institution. Of these, postoperative hemodynamic assessments using cardiac catheterizations were performed in 414 patients who had survived for at least 6 months after the operation. Our follow-up policy included a postoperative serial comprehensive assessment of the Fontan pathophysiology, including cardiac catheterizations, to improve the long-term outcome at 1 year postoperatively. Furthermore, the patients were followed-up every 5 years postoperatively (5). In December 1994, we started to assess the presence of PAVF using bubble contrast echocardiography as described below. We excluded 23 patients who had undergone Fontan and who had never been assessed for PAVF before December 1994. Thus, a total of 391 patients who had undergone Fontan were finally included in this study.

### Hemodynamics, Ventricular Function, and Brain Natriuretic Peptide

Cardiac catheterization using biplane cineventriculography was performed in all patients, except for 10 patients who were suspected of being allergic to contrast media. In the included patients, the hemodynamic variables measured included the central venous pressure (CVP, mmHg), cardiac index (CI, L/min/m^2^), pulmonary and systemic artery resistances (U•m^2^), morphological right and left ventricular volumes (using the Simpson’s rule), and ejection fraction (EF, %) (4). The end-diastolic ventricular volume was divided by the body surface area to obtain the end-diastolic volume index (EDVI, mL/m^2^). Atrioventricular valve regurgitation was estimated using color flow mapping and was graded as follows: none to mild, moderate, or severe during hospitalization. The plasma levels of brain natriuretic peptide (BNP) (pg/mL) were also measured.

### Assessment of Pulmonary Arteriovenous Fistulae

#### Diagnosis of Pulmonary Arteriovenous Fistulae and Veno-Venous Collaterals

At the end of catheterization, PAVF was diagnosed using bubble contrast echocardiography with the injection of approximately 3–5 mL of agitated saline solution, selectively into the right and left pulmonary arteries, and the contrast images were visualized using an equivalent of a standard apical four-chamber view (Vivid q scanner, GE Medical Systems Israel Ltd., Haifa, Israel). The magnitude (contrast in the functional systemic atrium and ventricle) was graded as non-mild, moderate, and severe (5, 6). Severe and moderate were considered diagnostic of PAVF. The presence of veno-venous collaterals (VVC) from any of the superior vena cava (SVC), inferior vena cava (IVC), and/or the innominate vein, to the pulmonary veins and functional left atrium, was also assessed by applying the same contrast echocardiography with an injection of agitated saline solution into these three great veins. The same grading of PAVF was applied to the VVC.

#### Unbalanced Pulmonary Artery Flow Distribution From the Hepatic Vein and Inferior Vena Cava

**FIGURE 1:**
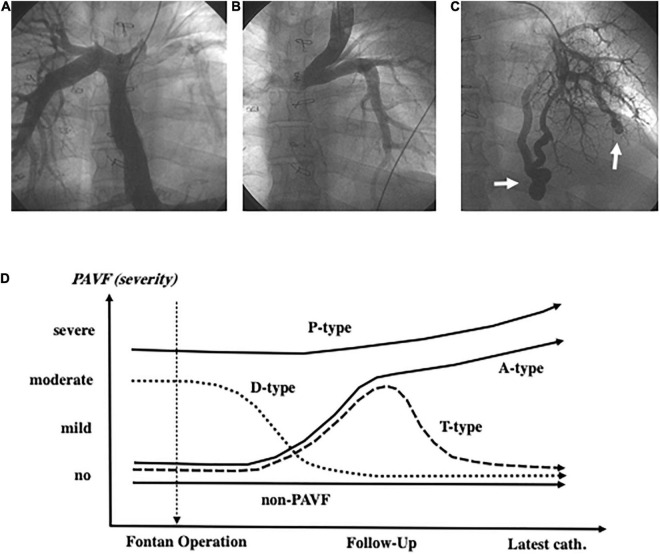
Angiography of the inferior vena cava shows an image of the very thin left pulmonary artery **(A)**. The left pulmonary artery is not visible because of diminished left pulmonary blood flow due to collision of blood flow between the left superior vena cava and the inferior vena cava **(B)**. Arrows indicate discrete-pulmonary arteriovenous fistulae **(C)**. PAVF classification based on the time course after the Fontan operation **(D)** and the definition of each type (A, T, D, P) is described in the “Materials and Methods” section. PAVF, pulmonary arteriovenous fistulae.

We evaluated the degree of unbalanced pulmonary artery flow distribution (PAFD) from the hepatic vein and IVC in 381 patients by performing angiography. Because of difficulties in grading the unbalanced PAFD, we judged it as positive when the right or the left pulmonary artery was not fully visible with a lack of ipsilateral peripheral artery images in our clinical conference as shown in Figures 1A–C.

#### Type of Pulmonary Arteriovenous Fistulae Based on the Morphology

We categorized our patients into three groups based on the morphology of the PAVF (i.e., diffuse-, discrete-, and non-PAVF), according to a previous study (4).

#### Type of Pulmonary Arteriovenous Fistulae Based on the Time-Course

We focused on patients who were diagnosed with PAVF at the latest Fontan evaluation (catheterization) and categorized these patients into four subgroups based on the time course after the Fontan operation: those with D-Type PAVF, which had disappeared within 1–5 years after the Fontan operation, were excluded from the final statistical analyses. The A-type PAVF, which had newly developed and continued after the operation. The P-Type PAVF was present before and continued after the operation. The T-type PAVF had newly developed and disappeared due to successful specific intervention (Figure 1D).

### Pulmonary Function and Cardiopulmonary Exercise Tests

Patients who had undergone Fontan also underwent a symptom-limited treadmill exercise using a ramp protocol and the peak oxygen uptake (VO_2_) along with the percutaneous oxygen saturation (SpO_2_) (PULSOX-Me300, Konica Minolta, Tokyo, Japan) throughout exercise testing. The exercise has induced a decline in SpO_2_ from rest to peak exercise was calculated (7) and the respiratory exchange ratio (= VCO_2_/VO_2_) at peak exercise of ≥1 was considered to be the peak effort of exercise performed in this study. We also measured vital capacity (VC; L), the percentage forced expiratory volume in 1 s (Spirosift SP-600, Fukuda Denshi, Tokyo), and VC was calculated as the percentage of the body height predicted normal value in our institute.

### Statistical Analysis

Because of skewed data distribution in the plasma BNP level, the logarithmic value was used for some subsequent statistical analysis. Data are expressed as means ± standard deviations (SD) or calculated as medians with interquartile ranges where appropriate. Differences in demographics, hemodynamics, pulmonary function, and cardiopulmonary exercise variables were analyzed using one-way ANOVA with Turkey’s *post hoc* test among the three groups. A univariate linear regression analysis was used to evaluate relationships between the continuous variables, and a multivariate linear regression analysis was used to detect the main correlates. Comparisons of prevalence of heterotaxy, medication usage, and type of surgical procedures were analyzed using chi-square or Fisher’s exact test. A status free from the onset of PAVF after the Fontan operation and death after the assessment of PAVF at the latest catheterization was estimated using the Kaplan–Meier method, and the differences in these event-free statuses between the groups were assessed using the log-rank tests. Cox’s proportional hazards model was used to predict the associations between clinical variables and the onset of PAVF or all-cause mortality. Variables that proved to be significant predictors of the outcome in the univariate analysis (*p* < 0.05) were included in the multivariate model to determine the independent predictors. All analyses were performed using the statistical software JMP 12 (SAS Institute, Cary, NC, United States). A two-sided *p*-value of <0.05 was considered statistically significant.

## Results

### Prevalence, Incidence, and Type of Pulmonary Arteriovenous Fistulae

**FIGURE 2:**
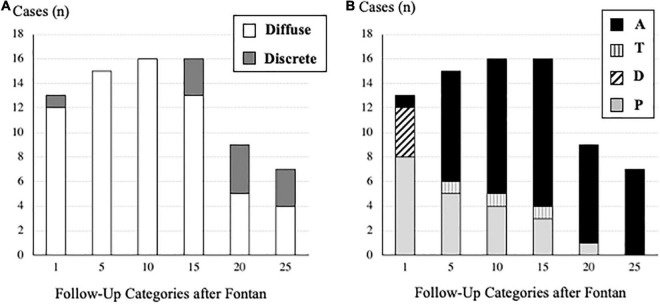
**(A)** Number of cases of pulmonary arteriovenous fistulae based on the morphology (diffuse or discrete) in each follow-up category after the Fontan operation. **(B)** Number of cases of pulmonary arteriovenous fistulae based on the time-course (A, T, D, P) in each follow-up category after the Fontan operation. Definition of each type (A, T, D, P) is described in the “Materials and Methods” section.

**FIGURE 3:**
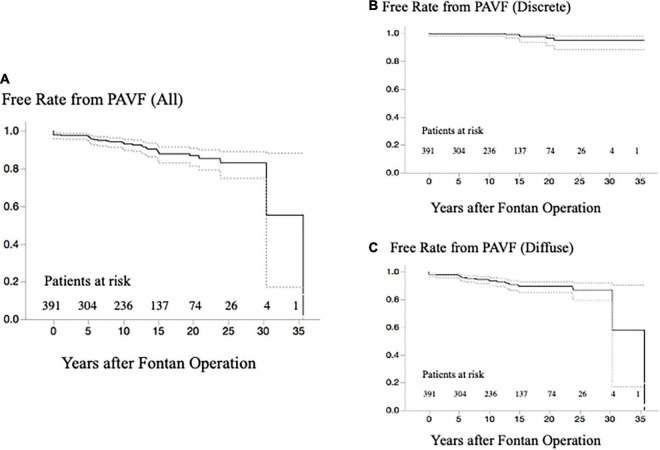
**(A)** Free rate from any PAVF after the last evaluation of PAVF at catheterization. **(B)** Free rate from any discrete-PAVF after the last evaluation of PAVF at catheterization. **(C)** Free rate from any diffuse-PAVF after the last evaluation of PAVF at catheterization. PAVF, pulmonary arteriovenous fistulae.

Concerning PAVF, the number of patients that we could evaluate at 1, 5, 10, 15, 20, and ≥25 years after Fontan operation were 391, 325, 259, 182, 107, and 53, respectively. After excluding four patients who had been diagnosed with D-Type PAVF, we identified a total of 36 patients with PAVF (9.2%), including 29 patients (80%) with diffuse PAVF, six patients (17%) with discrete PAVF, and one patient (3%) with diffuse and discrete PAVF. The latter was categorized as having diffuse PAVF because the dominant lesion was a diffuse PAVF. A comparison of clinical characteristics of patients with diffuse, discrete, and non-PAVF is presented in Table 1. Regarding the classification based on the serial time-course of PAVF, Type-A PAVF was the most frequent type (*n* = 25, 70%), followed by Type-P in eight (22%), and type-T in three (8%) patients. The prevalence and the types of PAVF (diffuse or discrete, A, D, T, or P) throughout the follow-up period are presented in Figures 2A,B. Type-D is presented in Figure 2A. When we assumed that the follow-up time was 0 in Type-P patients, the free rates from any PAVF, excluding Type-D, at 1, 5, 10, 15, 20, and ≥25 years after Fontan operation, were 98, 97, 94, 89, 87, and 83%, respectively (Figure 3A). The free rates from the diffuse or discrete PAVF are also presented in Figures 3B,C.

**TABLE 1:** Subject characteristics.

PAVF		Diffuse	Discrete	No	*p*
Cases (*n*)		30	6	355	
Age (year) at latest cath.		14 ± 10	22 ± 6	16 ± 10	0.1805
Male (%)		67	67	58	0.565
Age at 1st Fontan (year)		5 ± 6	8 ± 5	4 ± 5	0.0433
NYHA class		1.8 ± 0.8#	1.7 ± 0.5	1.3 ± 0.6	<0.0001
Heterotaxy (%)		47	67	26	0.0084
	Right Isomerism	10	33	21	0.3613
	Left Isomerism	37	33	5	<0.0001
SVtype (LV:%)		40	17	40	0.4734
Diagnosis (%)					
	UVH	27	17	23	
	TA	27	17	19	
	DORV	27	50	18	
	HLHS	3	0	5	
	Others	16	16	35	
Current Type of Repair (%)					0.0087
	APC	3	0	1	
	IAR	57	67	19	
	ECR	40	33	80	
Procedures prior Fontan (%)					
	APS	50	50	57	0.7492
	PAB	30	17	31	0.7348
	Glenn	47	33	65	0.051
	Fenestration	17	0	12	0.3551
Medications (%)					
	Diuretics	60	33	41	0.1299
	Anti-coaglant	77	83	75	0.8806
	ACEI/ARB	23	67	36	0.1019
	Beta blocker	17	33	26	0.4732
	Anti-arrhythmia	17	17	9	0.3598

*ACEI, angiotensin converting enzyme inhibitor; APC, atriopulmonary connection; APS, aortopulmonary shunt; ARB, angiotensin receptor blocker, connection; APS, aortopulmonary shunt; ARB, angiotensin receptor blocker; DORV, double outlet right ventricle; ECR, extracardiac rerouting; HLHS, hypolastic left heart syndrome; IAR, intraatrial rerouting; LV, left ventricle; NYHA, New York Heart Association; PAB, pulmonary artery banding; SV, systemic ventricle; TA, tricuspid valve atresia; UVH, univentricular heart. Values are mean ± SD. #Indicate p < 0.05 vs. group of Discrete and No, respectively.*

Among the patients who had undergone evaluation for the presence of PAVF, those who had undergone a complete evaluation of VVC (i.e., injections of agitated saline solution into the SVC, IVC, and innominate vein) were 100, 121, 153, 126, 78, and 48 at 1, 5, 10, 15, 20, and ≥25 years after the Fontan operation, respectively. The presence of VVC was confirmed in 74 (74%), 97 (80%), 97 (63%), 82 (65%), 50 (64%), and 26 (54%) patients, respectively; when patients with PAVF were separately analyzed, VVC at all postoperative stages was observed in 2 (100%), 6 (86%), 12 (100%), 11 (85%), 8 (100%), and 7 (100%) patients, respectively.

The SaO_2_ levels at each postoperative stage were significantly lower in patients with than in those without VVC (93 ± 4% vs. 95 ± 2%, 93 ± 3% vs. 95 ± 1%, 94 ± 3% vs. 95 ± 3%, 93 ± 3% vs. 95 ± 2%, 93 ± 6% vs. 96 ± 2%, and 92 ± 5% vs. 95 ± 2% at 1, 5, 10, 15, 20, and ≥25 years after the Fontan operation, respectively; *p* < 0.05).

### Unbalanced Pulmonary Artery Flow Distribution and Pulmonary Arteriovenous Fistulae

**FIGURE 4:**
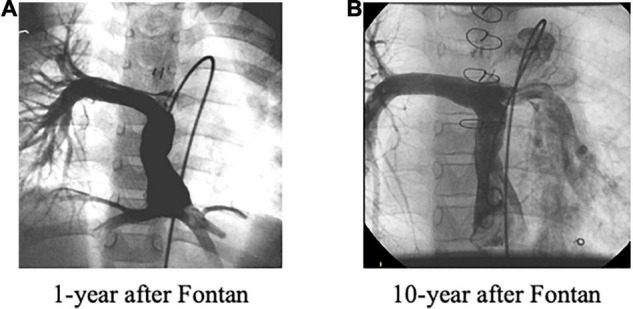
**(A)** Unbalanced pulmonary artery flow distribution at 1 year after the Fontan operation. **(B)** Improved unbalanced, i.e., “balanced,” pulmonary artery flow distribution was observed at 10 years after the Fontan operation due to significantly increased left pulmonary flow.

In our routine clinical conference, unbalanced PAFD from the hepatic vein and/or the IVC was identified in 16 (57%) out of 28 patients with diffuse PAVF, and in two (33%) out of six patients with discrete PAVF. In contrast, 26 (7.5%) out of 347 patients without PAVF were judged as having an unbalanced PAFD, which was much lower than the rate of unbalanced PAFD in patients without PAVF (*p* < 0.0001). None of the four D-type patients had unbalanced PAFD. Interestingly, seven of the 21 patients (33%) with Type-A PAVF, excluding two patients with liver cirrhosis and two patients who had undergone surgical redirection of the Fontan route, had shown significantly improved “balanced” PAFD when compared to the initial unbalanced PAFD at 1 year after the Fontan operation. A typical case is presented in Figures 4A,B.

### Comparison of Clinical Characteristics Based on the Pulmonary Arteriovenous Fistula*e* Morphology

#### Patient Characteristics

A summary of the comparisons is provided in Table 1. Compared to patients without PAVF, diffuse-PAVF showed greater NYHA class and a high prevalence of heterotaxy syndrome, especially in those with left isomerism (*p* < 0.0001). The same statistical trend was observed in patients with discrete-PAVF.

#### Hemodynamics and Cardiopulmonary Function

The comparisons of hemodynamic and cardiopulmonary variables among the three PAVF groups are summarized in Table 2. Compared to patients without PAVF, those with diffuse PAVF showed increased CVP, CI, and EDVI with lower SaO_2_, resembling a phenotype of high cardiac output heart failure. In contrast, the patients with discrete PAVF showed lower CI and high Rs, resembling a phenotype of low cardiac output heart failure, whereas there were no differences in the cardiac function, CVP, or SaO_2_ between the discrete- and non-PAVF groups. In addition, the SaO_2_ level for patients with diffuse, discrete, and non-PAVF after Fontan operation were 90 ± 8%, 93 ± 2%, and 94 ± 3% (*p* < 0.0001) at 1 year postoperatively; 91 ± 7%, 93 ± 1%, and 94 ± 3% (*p* < 0.0001) at 5 years postoperatively; 91 ± 7%, 94 ± 1%, and 95 ± 2% (*p* < 0.0001) at 10 years postoperatively; 91 ± 5%, 92 ± 2%, and 95 ± 2% (*p* < 0.0001) at 15 years postoperatively; 89 ± 11%, 94 ± 2%, and 95 ± 3% (*p* = 0.0011) at 20 years postoperatively; and 88 ± 9%, 93 ± 1%, and 94 ± 3% (*p* = 0.0007) at ≥25 years postoperatively, respectively. This also indicates significantly lower SaO_2_ levels only in patients with diffuse PAVF.

**TABLE 2:** Hemodynamics and exercise physiology in PAVF patients at the time of detection.

PAVF			Diffuse	Discrete	No	*p*
Cases (*n*)			30	6	355	
**Time course of PAVF**						
	A/P/T		20/7/3	5/1/0	–	
**Hemodynamics**						
	CVP (mmHg)		12 ± 3#	10 ± 3	10 ± 3	0.0003
	CI (L/min/m2)		3.3 ± 1.3†#	2.2 ± 0.4#	3.0 ± 0.6	0.0004
	Rp (U•m2)		1.5 ± 0.8	1.5 ± 0.5	1.3 ± 0.6	0.2566
	Rs (U•m2)		24 ± 12†	35 ± 9#	24 ± 7	0.0006
	SaO2 (%)		89 ± 9†#	93 ± 2	94 ± 3	<0.0001
	Hemoglobin (g/dl)		15 ± 2	16 ± 3	14 ± 2	0.0883
**Cardiac function**						
	EDVI (ml/m^2^)		94 ± 36#	89 ± 34	75 ± 25	0.0007
	EF (%)		56 ± 12	53 ± 8	55 ± 9	0.806
	AVVR ≥ mod (%)		11	0	9	0.5395
Log BNP (pg/mL)			2.9 ± 1.1	3.2 ± 0.6	2.8 ± 0.9	0.3646
Exercise Physiology (*n*)			(14)	(6)	(280)	
	Peak VO2 (% of N)		50 ± 15#	52 ± 13	59 ± 14	0.0081
	**SpO2 (%)**					
		Rest	92 ± 4#	91 ± 1#	94 ± 3	0.0007
		Peak	84 ± 7#	84 ± 3#	90 ± 5	<0.0001
		Decrease	−7 ± 5#	−7 ± 3	−4 ± 3	0.0002
	VE/VCO2 at peak (% of N)		134 ± 23†#	110 ± 22	115 ± 19	0.0001
Pulmonary function (*n*)			(14)	(6)	(250)	
	Vital Capacity (% of N)		73 ± 14	68 ± 12	80 ± 16	0.0547
	FEV1.0 (%)		87 ± 10	85 ± 8	87 ± 7	0.8501

*AVVR, atrioventricular valve regurgitation; BNP, brain natriuretic peptide; CI, cardiac index; CVP, central venous pressure; EF, ejection fraction of the systemic ventricle; EDVI, end-diastolic volume index of the systemic ventricle; FEV1.0, forced expired volume in one second; Rp, resistance of the pulmonary artery; Rs, resistance of the systemic artery; SaO2, arterial oxygen saturation; SpO2, percutaneous artery oxygen saturation; VE/VCO2, ventilatory equivalent for carbon dioxide output; VO2, oxygen uptake. Values are the mean ± SD. ^#,†^Indicate p < 0.05 vs. groups of No and Discrete, respectively.*

Regarding the cardiopulmonary variables, patients with diffuse-PAVF had the lowest peak VO_2_ and the highest VE/VCO_2_ with significantly low SpO_2_ during the exercise tests, including a greater SpO_2_ decline, compared to patients without PAVF (*p* < 0.001–0.0001).

### Specific Therapy for Pulmonary Arteriovenous Fistulae and All-Cause Mortality

#### Specific Therapy

Of the 36 patients with PAVF, no specific PAVF therapy, apart from oxygen inhalation, was used in 19 patients with diffuse PAVF and one patient with discrete PAVF. Catheter coil embolization was successfully used in all the remaining five patients with discrete PAVF. For the remaining 11 patients with diffuse PAVF, the therapies used included surgical redirection of the Fontan route in five patients, catheter balloon dilatation for stenosis between the Fontan route and the pulmonary artery with PAVF in three patients, multiple catheter coil embolization in two patients, and surgical redirection with additional catheter coil embolization in one patient with diffuse and discrete PAVF. Of these 16 patients who received specific therapies, 14 underwent the scheduled catheterization with an interval of 5 years. Of these, the overall SaO_2_ level did not change significantly (92 ± 3–90 ± 7%, *p* = 0.100), although SaO_2_ increased in seven patients (50%). SaO_2_ increased in all three patients with T-type PAVF, whereas it decreased in two P-type patients. In the three T-type patients, the diffuse-PAVF disappeared 5 years after the specific therapies, which included balloon dilatation for stenosis between the Fontan route and the right pulmonary artery in two patients, and surgical redirection of the Fontan route in one patient.

#### All-Cause Mortality

**FIGURE 5:**
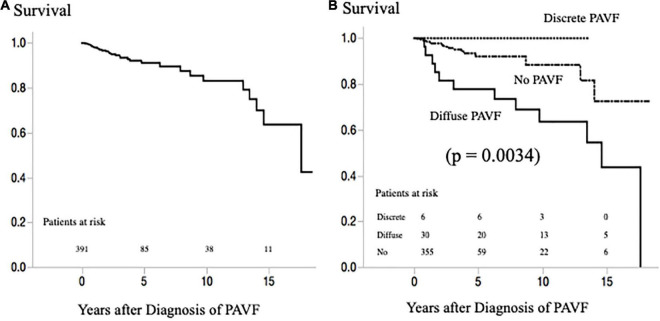
**(A)** Survival rate after latest evaluation of PAVF in all patients with a Fontan circulation. **(B)** Survival rate after latest evaluation of PAVF according to the morphology of PAVF. PAVF, pulmonary arteriovenous fistulae.

During a median follow-up period of 2.9 years after the last PAVF evaluation (15.9 years after the first Fontan operation), 31 patients died (due to heart failure in 11, sudden death in 6, cancer in 4, protein-losing enteropathy in 3, liver failure and stroke in 2 each, and due to other causes in 3 patients) (Figure 5A). Of the 36 patients with PAVF, no death occurred in patients with discrete-PAVF, and 12 patients with diffuse PAVF died (due to heart failure in 8, stroke in 2, and sudden death and liver failure in 1 patient each). Survivals based on PAVF morphology, after the latest evaluation, are presented in Figure 5B. Patients with diffuse-PAVF had a 3.6-fold higher risk of all-cause mortality than those without diffuse PAVF (hazard ratio [HR]: 3.60, 95% confidence interval: 1.59–7.81, *p* = 0.0026). Univariate Cox’s hazard model with hemodynamic variables revealed that high CVP, low SaO_2_, increased EDVI, low EF, and high plasma BNP levels were associated with a high risk of all-cause mortality (*p* < 0.05–0.0001). Of these hemodynamic variables and the presence of diffuse PAVF, the diffuse-PAVF, along with EF (*p* = 0.0145) and log BNP (*p* < 0.0001), were the independent predictors of all-cause mortality (HR: 3.57, 95% confidence interval: 1.14–9.80, *p* = 0.0181).

## Discussion

To our knowledge, this study is the first to report a comprehensive clinical profile of patients who had undergone Fontan with PAVF. Interestingly, we found the following: (1) we reconfirmed a close association between PAVF development and unbalanced PAFD; (2) the incidence of PAVF gradually increased as patients aged, although some cases of diffuse-PAVF disappeared after Fontan operation with balanced PAFD; (3) a high prevalence of VVC was found and VVC often coexisted with PAVF; (4) a specific therapy for PAVF was applied in half of the affected patients and long-term (5-year) efficacy (increase in SaO_2_) was observed in half of them; and (5) patients with diffuse-PAVF had a worse Fontan pathophysiology than those with discrete-PAVF or non-PAVF in terms of hemodynamics and all-cause mortality.

### Pulmonary Arteriovenous Fistulae and Hepatic Venous Flow

The “hepatic factor” hypothesis has been proposed to explain the mechanism of PAVF development after the Fontan operation; however, this factor has not been identified (2, 3, 8). In this study, we reconfirmed this phenomenon in our large cohort through visual evaluation, and liver cirrhosis was associated with bilateral diffuse-PAVF in two of our patients. Diffuse-PAVF may be one of the clinical phenotypes of Fontan-associated liver disease because of the high incidence of liver cirrhosis long after Fontan operation (9). Interestingly, diffused-PAVF disappeared with specific therapy immediately after diagnosis in three patients (T-type). PAVF may be inevitable after bidirectional cavopulmonary anastomosis (10), and early Fontan completion after this anastomosis may eliminate the possibility of developing PAVF (11). Especially, a much higher prevalence of D-type PAVF may be expected immediately after the Fontan operation when compared to the observed low prevalence of type-D (*n* = 4) at 1 year postoperatively.

We also confirmed a high prevalence of PAVF in patients with left isomerism who had undergone a Fontan operation (12). The high prevalence may be attributed mainly to the difficulties in equalizing hepatic flow distribution to the bilateral pulmonary arteries in cases where there is a small amount of hepatic flow compared to the greater systemic venous flow from the supra vena cava, which could easily produce streaming of the hepatic flow (11). In this regard, a similar Fontan route configuration with bidirectional cavopulmonary anastomosis may occur as these patients age and could lead to PAVF due to the consequent unbalanced stream of hepatic venous flow as demonstrated in our type-A patients.

### Unbalanced Pulmonary Artery Flow Distribution and Staging of Pulmonary Arteriovenous Fistulae

We should consider that the grade of unbalanced PAFD may depend on the stage of PAVF. In the early stages of PAVF where the pathophysiology may be reversible, the affected pulmonary artery receives low hepatic venous flow, thus, showing marked unbalanced PAFD. After this initial stage, ipsilateral pulmonary artery resistance gradually decreases as PAVF progresses and the ipsilateral pulmonary artery blood flow gradually increases. Finally, in the advanced stage of PAVF with even lower pulmonary artery resistance, where the pathophysiology may be irreversible, the affected pulmonary artery receives even more hepatic venous flow, showing an improved, i.e., “balanced” PAFD. We experienced seven such patients as described in Figure 4. This advanced PAVF further progresses to hypoxia even though the affected pulmonary artery can receive adequate “hepatic factor” because of the irreversible PAVF. This is why the degree of unbalanced PAFD does not always correlate with the presence of PAVF. In addition to these patients with “balanced” PAFD, our P-type patients also support this concept. The time interval from cavopulmonary bidirectional anastomosis, including Kawashima (*n* = 3) and Glenn (*n* = 3) procedures, to the Fontan operation, was 5.3 ± 3.7 years (range: 1.3–11 years), and this may have been long enough to establish advanced PAVF. As a result, surgical redirection was not effective although this intervention usually works shortly after the PAVF onset (13). Thus, it may be very important to recognize stage-dependent PAFD in patients with PAVF in terms of the diagnosis, as well as the appropriate therapy. In this regard, our three T-type patients did benefit from our follow-up policy.

Conversely, we encountered patients without PAVF with unbalanced PAFD, although the prevalence was low. We need to follow these patients carefully because they may tend to develop PAVF in the future, although we cannot deny another possible cause of PAVF other than “hepatic factor” (14).

### Impact of Pulmonary Arteriovenous Fistulae on Fontan Pathophysiology

Despite having no differences in PAFD or clinical background, there were distinct differences in the impact on Fontan pathophysiology between the diffuse- and discrete PAVF. Patients with diffuse-PAVF showed elevated CVP, high CI, and low SaO_2_ with greater EDVI, and had a high risk of all-cause mortality; all of which resembled the hemodynamic phenotype of Fontan failure with high cardiac output (5). In addition, liver cirrhosis may also be associated with a poor prognosis in patients with PAVF (15). However, no death occurred among our patients with discrete PAVF, including the patient with discrete and diffuse PAVF, despite their low cardiac output. The possibility of successfully performing a catheter intervention, as well as minimal influence on cardiovascular function may be associated with a better long-term outcome. Further studies may be required to clarify which factor(s), including genotypic factors, determine the morphological phenotype of PAVF (4).

### High Prevalence of Veno-Venous Collaterals

We confirmed the high prevalence of VVC (60–80%), especially in patients with PAVF (85–100%), with mild hypoxia, which indicates the difficulty of identifying incipient diffuse-PAVF with conventional diagnostic approaches, such as measuring arterial oxygen saturation or computed tomography (CT). Considering the importance of therapeutic timing of PAVF, the scheduled comprehensive assessment may be beneficial in some patients with PAVF similar to T-type patients, as demonstrated in this study.

### Study Limitations

This study had some limitations. First, unbalanced PAFD was evaluated visually in our clinical conference. A more objective approach, such as flow analysis using magnetic resonance imaging (MRI), may have been ideal, although PAFD depends on the PAVF stage. Second, in addition to underestimation of the overall prevalence of D-type PAVF, we could not exclude the possibility that our four D-type patients may have had T-type PAVF. Third, we did not check for the presence of a portosystemic shunt, which is another cause of PAVF, in all our patients. Fourth, because the incidence of PAVF may largely depend on the type of surgery applied, our results may not be generalized and should be interpreted with caution. Finally, we had no specific selection criteria that were used to identify candidates that required specific intervention. In our clinical conference, the therapeutic options were selected based on patients’ clinical conditions, the feasibility of catheter and/or surgical techniques, and the types of PAVF (morphology and time-course). Therefore, the efficacy of our specific management of PAVF may also not be generalized; therefore, large-scale prospective multicenter studies may be necessary for the future to standardize the management strategy of this unique Fontan-associated complication.

## Conclusion

We reconfirmed a close association between PAVF and unbalanced PAFD. The incidence of PAVF gradually increased as patients aged, although some diffuse-PAVF disappeared after the Fontan operation with balanced PAFD. The long-term efficacy of PAVF-specific therapy was observed in some patients. Our study is the first to report that patients with diffuse-PAVF had a worse Fontan pathophysiology than those with discrete-PAVF or non-PAVF in terms of hemodynamics and all-cause mortality.

## Data Availability Statement

The original contributions presented in the study are included in the article/supplementary material, further inquiries can be directed to the corresponding author.

## Ethics Statement

The studies involving human participants were reviewed and approved by Ethics Committee of the National Cardiovascular Center (M23-002-5). Written informed consent from the participants’ legal guardian/next of kin was not required to participate in this study in accordance with the national legislation and the institutional requirements.

## Author Contributions

HO designed the study, interpreted the data, and critically reviewed the manuscript. AM, KF, TI, HS, IS, and KK undertook data collection and critically reviewed the manuscript. MN contributed to data analyses, data interpretation, and critically reviewed the manuscript. All authors contributed to the article and approved the submitted version.

## Conflict of Interest

The authors declare that the research was conducted in the absence of any commercial or financial relationships that could be construed as a potential conflict of interest.

## Publisher’s Note

All claims expressed in this article are solely those of the authors and do not necessarily represent those of their affiliated organizations, or those of the publisher, the editors and the reviewers. Any product that may be evaluated in this article, or claim that may be made by its manufacturer, is not guaranteed or endorsed by the publisher.
